# Surgical outcomes of bridge-to-bridge therapy with extracorporeal left ventricular assist device for acute myocardial infarction in cardiogenic shock

**DOI:** 10.1186/s12872-022-02500-4

**Published:** 2022-02-16

**Authors:** Chiho Tokunaga, Atsushi Iguchi, Hiroyuki Nakajima, Fumiya Chubachi, Yuto Hori, Akitoshi Takazawa, Jun Hayashi, Toshihisa Asakura, Akihiro Yoshitake

**Affiliations:** Department of Cardiovascular Surgery, Saitama Medical University International Medical Center, 1397-1 Yamane, Hidaka-shi, Saitama 350-1298 Japan

**Keywords:** Extracorporeal left ventricular assist device, Acute myocardial infarction, Cardiogenic shock, Mechanical circulatory support, Heart transplant

## Abstract

**Background:**

Extracorporeal left ventricular assist device is often required for acute myocardial infarction patients in cardiogenic shock when temporary mechanical circulatory support fails to provide hemodynamic stabilization. This study aimed to evaluate the clinical outcomes of acute myocardial infarction patients in cardiogenic shock supported by an extracorporeal left ventricular assist device.

**Methods:**

This retrospective study enrolled 13 acute myocardial infarction patients in cardiogenic shock treated with an extracorporeal left ventricular assist device from April 2011 to July 2020.

**Results:**

Twelve (92.3%) and eleven (84.6%) patients were supported using venoarterial extracorporeal membrane oxygenation and intra-aortic balloon pumping before implantation, respectively. The median duration from acute myocardial infarction to extracorporeal left ventricular assist device implantation was 7 (3.5–24.5) days. The overall in-hospital mortality rate was 30.8% (n = 4). Extracorporeal left ventricular assist device was explanted in one patient for cardiac recovery; eight (61.5%) patients were approved as heart transplant candidates in whom the extracorporeal left ventricular assist device was exchanged for a durable left ventricular assist device; two (15.4%) expired while waiting for a heart transplant, and two (15.4%) received a successful transplant. The 1- and 3-year overall survival rates after extracorporeal left ventricular assist device implantation were 68.3% and 49.9%, respectively.

**Conclusions:**

The operative mortality after extracorporeal left ventricular assist device implantation in acute myocardial infarction patients in cardiogenic shock was favorable. Our strategy of early hemodynamic stabilization with extracorporeal left ventricular assist device implantation in these patients as a bridge-to-bridge therapy was effective in achieving better survival.

## Background

Cardiogenic shock is a complication that develops in 5%–10% of acute myocardial infarction (AMI) patients [1–3]. Furthermore, cardiogenic shock is one of the leading causes of mortality in AMI [4] and is associated with a 1-year mortality of up to 70–80% in AMI patients [2, 3]. Early revascularization is the primary treatment for improving the survival of AMI patients in cardiogenic shock [3]. Nevertheless, short-term mechanical circulatory support with an intra-aortic balloon pumping (IABP), venoarterial extracorporeal membrane oxygenation (VA-ECMO), or Impella® circulatory assist pump catheters (Abiomed Inc., Danvers, MA, USA) are often required to support hemodynamics in patients with an Interagency Registry for Mechanically Assisted Circulatory Support (INTERMACS) profile 1 status due to AMI [5]. AMI in cardiogenic shock is one of the most challenging diseases despite recent medical advances, and when these temporary mechanical circulatory support systems fail to provide hemodynamic stabilization, patients are considered candidates for extracorporeal left ventricular assist device (LVAD) implantation as this device provides stronger systemic flow support and improves the left ventricular unloading. Recently, our strategy has shifted towards an early administration of extracorporeal LVAD implantation for maximum circulatory support for circulatory failing patients under a temporary mechanical circulatory support to avoid the development of end-organ dysfunction among AMI patients in cardiogenic shock.

This study aimed to evaluate the clinical characteristics and outcomes of patients on extracorporeal LVAD support for AMI in cardiogenic shock as a bridge-to-decision, bridge-to-recovery, or bridge-to-bridge therapy.

## Materials and methods

### Patients

From April 2011 to July 2020, a total of 43 patients underwent extracorporeal LVAD implantation for cardiogenic shock or acute decompensated heart failure (INTERMACS profile 1 or 2 status) at the international medical center in Saitama Medical University. Of these patients, 13 (eight males, 61.5%) were treated for AMI in cardiogenic shock and were the focus of  this study. The patient’s clinical data were retrospectively extracted from their medical records. Primary diagnoses other than AMI leading to the implantation of an extracorporeal LVAD were dilated cardiomyopathy (n = 16), fulminant myocarditis (n = 10), and others (n = 4). Cardiogenic shock was defined as a systolic blood pressure of < 90 mm Hg in the absence of hypovolemia or any sign of clinical hypoperfusion [6].

### Ethical approval

This study was approved by the institutional review board of Saitama Medical University (approval no. 18-263), and the requirement for informed consent was waived owing to the retrospective nature of the study.

### Operative indications and techniques

**Fig. 1:**
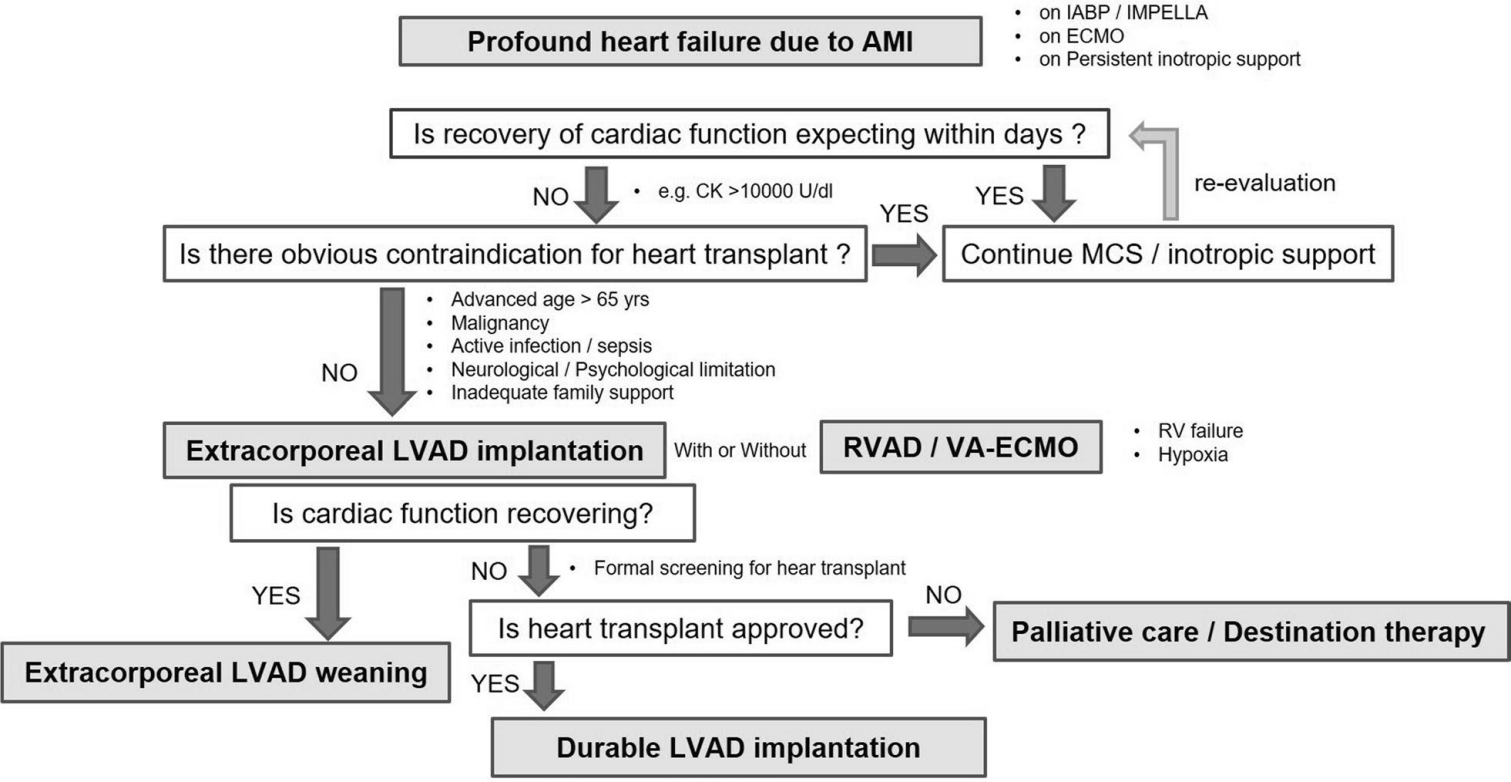
Outline of our strategy for extracorporeal LVAD implantation in AMI patients in cardiogenic shock. LVAD, left ventricular assist device; AMI, acute myocardial infarction; RVAD, right ventricular assist device; VA-ECMO, venoarterial extracorporeal membrane oxygenation

Usually, either a cardiologist or a cardiac surgeon was the first point of contact according to the discretion of the referring hospital. After diagnosing the patients with AMI in cardiogenic shock under inotropic support and temporary mechanical circulatory support, including IABP, percutaneous VA-ECMO, or Impella®, and if patients demonstrated sustained hypotension under a temporary mechanical circulatory support, along with a guideline-oriented maximum medical treatment for heart failure, a multidisciplinary LVAD team was consulted. Extracorporeal LVAD implantation was considered to prevent further progression of end-organ dysfunction and development of complications associated with temporary mechanical circulatory support if sufficient cardiac function recovery appeared unlikely within a short time. Clinical information of the patients was shared, and the LVAD team rapidly investigated the presence of contraindications for a heart transplant, including advanced age > 65 years old, persistent end-organ dysfunction, preoperative life-limiting comorbidities, or a lack of familial or financial support. Regarding end-organ dysfunction, preoperative temporary hemodialysis was not in itself a contraindication for extracorporeal LVAD implantation at our institute. In the absence of obvious contraindications for a heart transplant, the LVAD team proceeded with extracorporeal LVAD implantation as a bridge-to-decision, bridge-to-recovery, or bridge-to-bridge therapy. Our strategy is outlined in Fig. 1.

Surgery was initiated via a median sternotomy approach. The procedure was performed under cardiopulmonary bypass without cardiac arrest if there were no concomitant procedures. The left ventricular apex was chosen as the site for inflow cannula insertion. Large horizontal mattress sutures (2–0 non-absorbable) were placed through the full thickness of the left ventricular myocardium. If the left ventricular myocardium was too fragile for a direct suture, three pieces of endocardial fan-shaped Teflon felt strips were inserted inside the left ventricle to reinforce the myocardium.

If the cardiac function was not recovered after extracorporeal LVAD implantation, patients were formally evaluated for a heart transplant. When the patients met the criteria and were approved as heart transplant candidates by the Heart Transplant Candidate Registry Committee of the Japanese Circulation Society, extracorporeal LVAD was exchanged for a durable LVAD as a bridge-to-transplant therapy.

### Data collection

All data were extracted from the medical records, and follow-up data were collected until April 2021. Model of End-Stage Liver Disease-eXcluding International Normalized Ratio (MELD-XI) scores were calculated for assessing liver dysfunction in heart failure using the following formula: MELD-XI score = 5.11 × Log (bilirubin mg/dL) + 11.76 × Log (creatinine mg/dL). Laboratory values <1 were assigned a value of 1 in the calculation to avoid results with negative values [7, 8].

### Statistical analysis

Normally distributed continuous variables were expressed as mean ± standard deviation, whereas continuous variables with skewed distribution were presented as a median with interquartile range. The Kaplan–Meier method was employed for calculating the long-term survival. All data were analyzed using SPSS version 24.0 (IBM Corp., Armonk, NY, USA).

## Results

### Patient characteristics

The patient’s preoperative demographic data are presented in Table 1. The mean age of the patients was 45.5 ± 13.4 (range, 18–63) years, and 8 (61.5%) of the 13 patients were < 50 years old. The peak creatinine kinase and creatine kinase-myocardial band levels were 14,644 ± 10,141 IU/L and 550 ± 329 IU/L, respectively. In six patients, the peak creatine kinase level was > 10,000 IU/L.

**Table 1:** Preoperative demographic data of patients

Variables	N = 13
Male sex	8 (61.5%)
Mean age in years (range)	45.5 ± 13.4 (18–63)
*Co-morbidities*	
Hypertension	5 (38.5%)
Hyperlipidemia	4 (30.8%)
Diabetes mellitus	6 (46.2%)
Cerebrovascular disease	0
Acute kidney injury	10 (76.9%)
Renal replacement therapy	4 (30.8%)
Chronic atrial fibrillation	0
Paroxysmal atrial fibrillation	2 (15.4%)
LVDd (mm)	55.7 ± 8.7
LVDs (mm)	49.0 ± 8.7
EF (%)	16.0 ± 4.2
on IABF	11 (84.6%)
on VA-ECMO	12 (92.3%)
INTERMACS Profile 1	13 (100%)

Value given as number (%) or mean ± SD (range)

LVDd, left ventricular diastolic diameter; LVDs, left ventricular systolic diameter; VA-ECMO, venoarterial extracorporeal membrane oxygenation; INTERMACS, Interagency Registry for Mechanically Assisted Circulatory Support; EF, ejection fraction; IABP, intra-aortic balloon pump

The preoperative serum creatinine level was 1.5 ± 0.8 mg/dL, and four (30.8%) patients required temporary renal replacement therapy preoperatively. The renal dysfunction in these patients was reversible, and all four were successfully bridged to durable LVAD implantation.

The MELD-XI scores before and 2 months after implantation of extracorporeal LVAD significantly improved from 17.2 ± 6.5 to 10.0 ± 1.8 (*p* < 0.05).

In this study, 12 (92.3%) and 11 (84.6%) patients received VA-ECMO and IABP support, respectively, before considering extracorporeal LVAD implantation, whereas none of the patients were supported with Impella®. At our institute, four patients were treated for cardiogenic shock due to AMI after approving Impella in 2018, two of whom underwent left main tract (LMT) dissection due to acute aortic dissection, which was a contraindication for Impella® use. The other two patients had a broad myocardial infarction with an increased creatine kinase level > 10,000 IU/L. We considered that their cardiac function was unlikely to recover under percutaneous mechanical circulatory support using Impella® within weeks due to broad damage of the left ventricular myocardium. Therefore, Impella® was not used in any of these patients. One patient who experienced heart failure relapsed after being weaned from the VA-ECMO underwent IABP insertion. All patients were considered to have an INTERMACS profile 1 status, as they were all managed with temporary mechanical circulatory support for hemodynamic stabilization. The median duration from the onset of AMI to extracorporeal LVAD implantation was 7 (3.5–24.5) days. The median duration from consultation with the LVAD team to extracorporeal LVAD implantation was 3 (0–10.5) days. Moreover, six (46.2%) patients underwent extracorporeal LVAD implantation within 24 h of consultation.

As shown in Table 2, the primary etiologies of AMI were atherosclerotic diseases in seven (53.8%) patients, LMT occlusion due to acute aortic dissection in two (15.4%), spontaneous coronary artery dissection in two (15.4%), LMT stenosis due to Takayasu arteritis in one (7.7%), and post-Bentall operation in one (7.7%). The culprit coronary lesion of AMI was the LMT in nine (69.2%) patients and the proximal left anterior descending artery in the other four (30.8%). Notably, nearly half of the patients had AMI due to non-atherosclerotic diseases at a young age. The mean age of patients who had the atherosclerotic disease as the primary etiology was 50.3 years and that of the others with a non-atherosclerotic etiology was 39.7 ± 15.1 years. However, the difference was not significant.

**Table 2:** Primary etiologies of AMI

Primary etiology of AMI	N = 13
Atherosclerosis	7 (53.8%)
AAD with LMT dissection	2 (15.4%)
p-Bentall LMT stenosis	1 (7.7%)
LMT stenosis due to Takayasu arteritis	1 (7.7%)
Spontaneous coronary artery dissection	2 (15.4%)

Value given as number (%)

AMI, acute myocardial infarction; AAD, acute aortic dissection; LMT, left main tract

For coronary revascularization before extracorporeal LVAD implantation, percutaneous coronary intervention was performed in nine (69.2%) patients, coronary artery bypass grafting (CABG) in three (23.1%), and LMT repair during acute aortic dissection surgery in one (7.7%).

### Procedures of extracorporeal LVAD implantation

During extracorporeal LVAD implantation, centrifugal pumps (six Gyro pumps [Medtronic Bio-Medicus, Minneapolis, MN, USA] and three MERA centrifugal blood pumps [Senko Medical Instrument Mfg. Co. Ltd., Tokyo, Japan]) were predominantly used in nine (69.2%) patients, whereas a pulsatile pump (VAS; NIPRO Corporation, Osaka, Japan) was utilized in the remaining four (30.7%). In one patient, an oxygenator (Senko Medical Instrument Mfg. Co. Ltd., Tokyo, Japan) was spliced into the LVAD circuit for a severe hypoxemic respiratory failure due to the left heart failure. We considered that the centrifugal pump was more effective in supporting and controlling systemic perfusion than the pulsatile pump. Therefore, since 2015, the centrifugal pump has been more commonly utilized in patients with body weight > 70 kg, in whom the pulsatile pump was unlikely to achieve sufficient flow. The mean duration of the centrifugal pump support to durable LVAD implantation was 101.7 ± 39.8 days. Patients under extracorporeal LVAD support were cared for in the hospital and encouraged to undergo physical rehabilitation, including ambulation, for further heart transplant approval.

As a concomitant procedure with extracorporeal LVAD implantation, CABG was performed in three patients, and a right ventricular assist device was implanted in one patient. The median duration from cardiogenic shock due to AMI to extracorporeal LVAD implantation was 7 days (Table 3).

**Table 3:** Operative characteristics of extracorporeal LVAD implantation

Operative characteristics of eLVAD	N = 13
Duration from AMI to eLVAD implantation (days)	7.0 (3.5–24.5)
*Type of eLVAD*	
NIPRO	4
Gyro	6
MERA	3
Concomitant procedure	4 (30.8%)
CABG	3 (23.1%)
ECMO (RVAD)	1 (7.7)

Value given as number (%) or median (interquartile range)

eLVAD, extracorporeal left ventricular assist device; AMI, acute myocardial infarction; RVAD, right ventricular assist device; CABG, coronary artery bypass grafting; ECMO, extracorporeal membrane oxygenation

### Clinical outcomes

The patient’s early and late surgical outcomes are outlined in Table 4. The mean follow-up period was 685.2 ± 582.7 days. Of the 13 patients with extracorporeal LVAD, eight (61.5%) were evaluated for heart transplant as their cardiac function did not recover. Once patients were approved as heart transplant candidates by the Heart Transplant Candidate Registry Committee of the Japanese Circulation Society, extracorporeal LVAD was converted to durable LVAD as a bridge-to-transplant therapy. All eight patients were discharged after durable LVAD implantation. The median duration from extracorporeal LVAD to durable LVAD was 90.0 (75.0–129.0) days. Two patients required more than 120 days to confirm their candidacy for heart transplantation.

**Table 4:** Surgical outcomes of extracorporeal left ventricular assist device implantation for acute myocardial infarction in cardiogenic shock

Surgical outcomes of eLVAD	N = 13
eLVAD explant for recovery	1 (7.7%)
Morbidity	
Re-exploration for bleeding	1 (7.7%)
Bleeding from inflow site	0
In-hospital mortality	4 (30.8%)
Intracranial hemorrhage	3
Intraperitoneal hemorrhage	1
Implantable LVAD (iLVAD) upgrade	8 (61.5%)
Duraheart	3
Heartmate II	3
Jervilk 2000	2
Duration from eLVAD to iLVAD implantation (days)	90 (75–129)
Discharge home	8 (61.5%)

Value given as number (%) or median (interquartile range)

Of the remaining five patients who did not apply for a transplant, one was weaned from an extracorporeal LVAD after 298 days of support, whereas the other four expired. The overall in-hospital mortality rate for AMI in cardiogenic shock was 30.8%. The deaths were due to intracranial hemorrhage in three patients and intraperitoneal hemorrhage in one. The mean length of hospital stay for AMI in cardiogenic shock was 175.6 ± 94.3 days. Regarding operative morbidity, two patients required re-exploration for postoperative bleeding; however, no bleeding was observed from the inflow cannulation site.

**Fig. 2:**
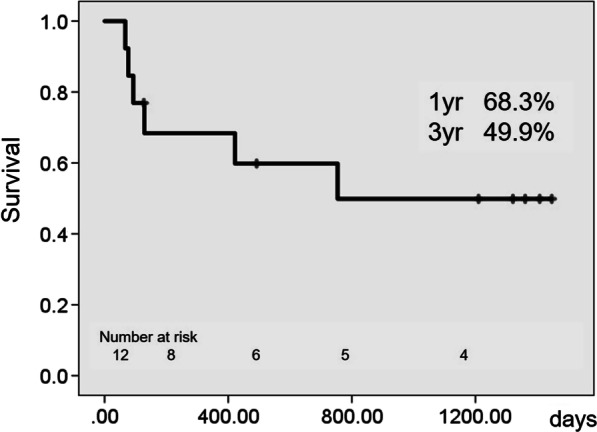
Overall survival after extracorporeal LVAD implantation in AMI patients in cardiogenic shock. AMI, acute myocardial infarction; LVAD, left ventricular assist device

Of the eight patients with durable LVAD, two expired while waiting for a heart transplant: one patient expired due to intracranial hemorrhage at 345 days, whereas the other expired suddenly of an unknown cause 625 days after durable LVAD implantation. Two patients underwent successful heart transplantation after 1447 and 1156 days, respectively. The bridge-to-transplant success rate was 15.4%. The 1- and 3-year overall survival rates after extracorporeal LVAD implantation in AMI patients with severe cardiogenic shock were 68.3% and 49.9%, respectively (Fig. 2).

## Discussion

This study primarily observed that extracorporeal LVAD implantation for AMI in cardiogenic shock, as in patients with an INTERMACS profile 1 status, was useful in providing hemodynamic stabilization and helped optimize end-organ dysfunction before obtaining candidacy for heart transplantation as a bridge-to-recovery, bridge-to-decision, or bridge-to-bridge therapy. Furthermore, two patients successfully underwent heart transplantation after extracorporeal LVAD implantation.

Cardiogenic shock secondary to AMI is the leading cause of mortality in AMI [1–3, 9]. The initiation of temporary mechanical circulatory support, including IABP, VA-ECMO, or temporary percutaneous circulatory support devices, such as Impella®, for hemodynamic maintenance followed by emergent coronary revascularization either by percutaneous coronary intervention or CABG, is essential for AMI patients in cardiogenic shock [10, 11]. However, no randomized controlled trials that investigated the value of temporary mechanical circulatory support in cardiogenic shock have demonstrated its survival benefits yet [11, 12].

LVAD implantation has already become an established treatment for end-stage heart failure. The most common indication for LVAD implantation is a bridge-to-heart transplant [10, 11, 13, 14]. Recently, the implementation of LVAD implantation has expanded into more acute and critical settings with favorable outcomes when guideline-directed medical therapy and conventional percutaneous mechanical circulatory support failed to provide hemodynamic maintenance [9, 15, 16].

The indications for primary durable LVAD implantation are limited to its use as a bridge for heart transplant by Japan’s national health insurance reimbursement system, and it is only applied to heart transplant candidates [17, 18]. Moreover, primary durable LVAD implantation is not recommended for patients with an INTERMACS profile 1 status. The higher cost of durable LVAD devices brings this strict policy to opt for a primary durable LVAD implantation. Therefore, extracorporeal LVAD is more commonly used in Japan compared with other countries. According to the annual report from the Japanese registry for mechanical assisted circulatory support in 2020, extracorporeal LVAD was implanted in 12.3% of total LVAD implantation cases [19].

Because of this background, it is mandatory to assess the indications of a heart transplant from both medical and social points of view before opting for a durable LVAD implantation for AMI patients in cardiogenic shock. However, assessing the reversibility of end-organ dysfunction in patients under conventional temporary mechanical circulatory support, as in those with INTERMACS profile 1 status, is often difficult owing to hemodynamic instability.

In this study, 70% of the patients had broad AMI involving LMT lesion, and > 80% of the patients were already under temporary mechanical circulatory support to survive but still demonstrated hemodynamic instability and end-organ dysfunction. Therefore, extracorporeal LVAD implantation was chosen for a more advanced hemodynamic support to prevent further end-organ dysfunction. After providing adequate flow support by extracorporeal LVAD implantation, the renal dysfunction requiring preoperative hemodialysis was reversed, and the MELD-XI scores representing liver function were improved in our study.

Additionally, most AMI patients in cardiogenic shock require several weeks or longer to evaluate their candidacy for a heart transplant. Since longer circulatory support by conventional mechanical circulatory support increases the risk of complications, including infection, bleeding, and malperfusion related to its access, the duration of its use is limited. Advanced hemodynamic flow support with extracorporeal LVAD is expected to provide time, and aid in optimizing the organ function for an accurate evaluation of heart transplant candidacy in this critical situation before durable LVAD implantation.

In this study, we demonstrated an in-hospital mortality rate of 30.8% with extracorporeal LVAD implantation in AMI patients in cardiogenic shock, which was comparable to the in-hospital mortality rates of 24%–33% indicated by studies conducted in Western countries [9, 15, 20]. As this procedure was applied to patients in cardiogenic shock despite being under temporary mechanical circulatory support, the operative mortality after extracorporeal LVAD implantation in AMI patients in cardiogenic shock was considered favorable but in definite need of improvement.

The optimal management of patients in cardiogenic shock remains debated with various approaches and reported outcomes. It is well known that retrograde ECMO flow increases end-diastolic pressure of left ventricle and exacerbating pulmonary congestion. McCarthy et al. reported that ECMO before LVAD implantation is associated with poor survival [21]. Therefore, when a sign of organ dysfunction was observed under VA-ECMO support, we believe that rapid conversion of VA-ECMO to more advanced mechanical circulatory support including extracorporeal LVAD is essential. In addition, recent research focuses on patients in cardiogenic shock due to AMI receiving percutaneous mechanical circulatory support using Impella®. The use of Impella® was approved in Japan in 2017, and its utilization rate has rapidly increased owing to its value in supporting the rate of systemic perfusion and left ventricular unloading [22]. A few studies have demonstrated the beneficial effect of Impella® on cardiac hemodynamics [23, 24]; Schafer et al. in their observational study reported that the implantation of Impella® before percutaneous coronary intervention in AMI patients in cardiogenic shock was associated with lower mortality [25]. In recent medical treatment progress, the Impella® mechanical circulatory support device is important for the primary treatment of cardiogenic shock due to AMI, but still has some limitations, including the amount of flow support, duration of use, and access. Therefore, we believe that extracorporeal LVAD is still an imperative therapeutic option for cardiogenic shock and could be utilized for appropriately selected patients as we have reported in this study.

In this study, most of the patients required extracorporeal LVAD implantation from the left ventricular apex within 7 days of AMI onset. Left ventricular disruption upon inflow cannulation is one of the concerns in AMI patients with fragile ventricular myocardium. Nonetheless, Pawale et al. reported that apical cannulation can be safely applied in AMI patients [26]. We placed sutures through the full thickness of the myocardium using an endocardial Teflon felt strip insertion to reinforce the cannulation site if the left ventricular myocardium was fragile. Our technique appears effective in securing the inflow cannulation site as no re-exploration was required related to bleeding from the inflow cannulation site.

Among other concerns, our strategy is at risk of repeated sternotomy for the explantation of extracorporeal LVAD and implantation of durable LVAD. Recently, a less invasive LVAD implantation technique with a left mini-thoracotomy and femoral venous cannulation as inflows and right axillary artery cannulation as an outflow was reported [27]. A less invasive surgical approach has the advantage of reducing blood loss and surgical trauma and might lead to faster patient recovery. However, there is a potential risk of the inability to control bleeding because of the limited visual field. As we believe that precise hemostasis is essential for the surgical success of this complicated procedure, we consider median sternotomy as the appropriate approach for this strategy.

Extracorporeal LVAD was converted to durable LVAD as a bridge-to-transplant therapy without major adverse events in 8 of 13 patients. All patients were discharged and were able to return to their social activities as office workers, homemakers, or students, as their functional capacity improved after durable LVAD implantation. Furthermore, two of the eight patients with durable LVAD successfully underwent heart transplantation.

The findings of this study indicated that our strategy of early hemodynamic stabilization with extracorporeal LVAD implantation in AMI patients in cardiogenic shock, followed by conversion to durable LVAD implantation as a bridge-to-transplant therapy, was effective in achieving better survival and further improvements in the patient’s quality of life. In addition, a relatively young population with the primary etiology of non-atherosclerotic diseases might have contributed to our results.

Dang et al. reported that the 1-year overall survival after LVAD implantation in AMI patients in cardiogenic shock was 63.5% [16]. Pawale et al. reported that the 1-year overall survival after direct durable LVAD implantation in AMI patients in cardiogenic shock was 73% [26]. In our study, the 1- and 3-year overall survival rates after extracorporeal LVAD implantation in AMI patients in cardiogenic shock were 68.3% and 49.9%, respectively. Despite seriousness of the conditions of the patients in this study and the differences in the background of the Japanese indications for durable LVAD implantation with a longer waiting period of approximately 900 days for a heart transplant in Japan [28], we consider our data to be acceptable. Once the patient’s condition has been stabilized with an extracorporeal LVAD implantation before the occurrence of irreversible progression of end-organ dysfunction and can survive the cardiogenic shock, their mid-term survival after durable LVAD implantation as a bridge-to-transplant therapy is expected to be the same as that reported by studies from other countries on AMI patients in cardiogenic shock [16, 26].

In contrast, the cost-effectiveness of primary extracorporeal LVAD implantation compared with durable LVAD implantation for critically ill patients with an IMTERMACS profile 1 is an important clinical question. The answer depends on the early survival rate of extracorporeal LVAD implantation, the success rate of bridge-to-bridge therapy, and longer quality-of-life quantification towards heart transplantation. The estimated total cost of bridge-to-bridge therapy using extracorporeal and durable LVAD devices could reach more than $300,000 in our country. Although, AMI in cardiogenic shock developed in relatively young and socially active patients in this cohort, and we believe that our strategy is still beneficial for survival in appropriately selected patients. Further investigations are necessary to conclude this issue.

### Study limitations

This study has several limitations. First, it was a single-center retrospective design with small sample size. Second, Impella® was not used in any of our participants, although it was gaining favor as an important primary treatment strategy for cardiogenic shock due to AMI. Finally, the reproducibility of our results might vary, and they might not be extendable to the entire population of AMI patients in cardiogenic shock.

Nevertheless, few studies have focused on extracorporeal LVAD implantation in AMI patients in cardiogenic shock, followed by durable LVAD implantation for a long period before undergoing heart transplantation. Our experience supports the use of extracorporeal LVAD implantation as an important therapeutic option for appropriately selected AMI patients in cardiogenic shock.

Further studies on this patient population are necessary to elucidate the benefits and risks of extracorporeal LVAD implantation in AMI patients in cardiogenic shock as a bridge-to-recovery, bridge-to-decision, or bridge-to-bridge therapy.

## Conclusion

Our strategy of extracorporeal LVAD implantation for AMI in cardiogenic shock was useful in providing hemodynamic stabilization and optimizing end-organ dysfunction before obtaining candidacy for heart transplantation as a bridge-to-recovery, bridge-to-decision, or bridge-to-bridge therapy in selected patients. Determining the appropriate timing of extracorporeal LVAD implantation for preventing further end-organ dysfunction is essential in improving the mortality and morbidity rates in AMI patients in cardiogenic shock.

## Data Availability

The data underlying this article cannot be shared publicly due to the privacy of individuals that participated in the study. The data will be shared on reasonable request to the corresponding author.

## Abbreviations

AMI: Acute myocardial infarction
IABP: Intra-aortic balloon pumping
VA-ECMO: Venoarterial extracorporeal membrane oxygenation
ITERMACS: Interagency registry for mechanically assisted circulatory support
LVAD: Left ventricular assist device
MELD-XI: Model of End-Stage Liver Disease-eXcluding International Normalized Ratio
LMT: Left main tract
CABG: Coronary artery bypass grafting
